# Behavioral and psychosocial correlates of fasting blood glucose in a resource-constrained diabetes clinic in the Dominican Republic: an exploratory network analysis

**DOI:** 10.3389/fcdhc.2026.1978637

**Published:** 2026-10-07

**Authors:** Amerigo Rossi, Mónica O. Rossi, Aurelina C. Jiménez, Ali Boolani

**Affiliations:** 1 Interdisciplinary Health Sciences, New York Institute of Technology, Old Westbury, NY, United States; 2 Health and Nutrition Sciences, Brooklyn College, New York, NY, United States; 3 Centro Regional Contra la Diabetes, Nagua, Dominican Republic; 4 Human Performance and Nutrition Research, Oklahoma State University, Stillwater, OK, United States

**Keywords:** Dominican Republic, fasting blood glucose, health inequalities, network analysis, physical activity, psychosocial factors, type 2 diabetes

## Abstract

**Objective:**

Examine the associations between demographic, behavioral, and psychosocial characteristics and fasting blood glucose among adults with type 2 diabetes (T2D) at a regional diabetes clinic in the Dominican Republic (DR).

**Methods:**

This cross-sectional study enrolled 65 adults with T2D who received care from a non-profit regional diabetes clinic in the DR. Participants completed interviewer-administered questionnaires to assess sleep quality, perceived stress, diet quality, health-related quality-of-life, and physical activity (PA). Fasting blood glucose was measured using capillary testing by a clinic nurse. A complete-case network analysis was conducted to examine conditional associations among study variables.

**Results:**

Only 15 participants (23%) had fasting blood glucose within the 80–130 mg•dL^-1^ target range for people with T2D, with significantly higher levels among men (243 ± 93 vs. 170 ± 64 mg•dL^-1^, p<0.001). Participants reported poor sleep quality (98%), moderate-to-high stress (97%), and low consumption of fruits and vegetables. Although 95% of participants met or exceeded recommended PA volume, activity was primarily from occupational and household tasks performed at light-to-moderate intensity. PA was the most connected variable within the network, and was inversely related to fasting blood glucose.

**Conclusions:**

In this resource-constrained DR clinic sample, poor fasting blood glucose coexisted with substantial sleep, stress, and diet deficiencies, despite substantial low-to-moderate-intensity PA. The network findings support diabetes self-management interventions to improve physical activity, sleep hygiene, stress reduction, and diet education, to improve glycemic control in low- and middle-income countries. Future prospective network analyses may identify context-specific behavioral targets for improving glycemic control in similar populations.

## Introduction

Type 2 Diabetes Mellitus (T2D) is a chronic illness that affects the body’s ability to process blood glucose, leading to significantly higher risk of morbidity and premature mortality (1). Over 500 million people are currently living with T2D globally, and that number is expected to rise to nearly 800 million by 2050. Urban areas have nearly 50% higher prevalence than rural areas, and middle-income countries are expected to have nearly double the prevalence growth rate (21%) compared to high- (12%) and low-income (12%) countries (2).

Across all low- and middle-income countries (LMIC), fewer than 5% of people with diabetes receive all recommended treatments (3) which limits treatment effectiveness. More research is needed to evaluate diabetes care programs in LMIC to develop evidence-based programming at diabetes care centers (5). The Dominican Republic (DR) is a middle-income country in which the prevalence of diabetes has increased from 3% in 2000 to 18% of the adult population in 2024 (4). Despite the rising prevalence, one study in rural DR found that only 51% of the sample had ever heard of diabetes and 72% of participants T2D had been undiagnosed (5). A comprehensive intervention based on the Chronic Care Model across two rural diabetes clinics in the DR reduced glycated hemoglobin (HbA1c) by 0.13% per year (6), indicating the potential for effective treatments despite low disease recognition.

To develop evidence-based practices, it is important to explore the prevalence of T2D behavioral and psychosocial correlates among the patient population. Despite growing T2D in DR and other LMIC, little is known about the relative contribution of various behavioral and psychosocial correlates for fasting blood glucose among people receiving care for T2D. At the *Centro Regional Contra la Diabetes Nagua* (Nagua Regional Diabetes Center; NRDC), which serves a primarily urban, resource-constrained population along the north coast of DR, people with T2D have very high levels of physical activity (7), indicating that other lifestyle factors may be important for complementary diabetes treatment (8). Therefore, further inquiry is warranted to determine which behavioral and psychosocial correlates are critical for developing effective comprehensive lifestyle interventions in this population.

Network analyses can be used to examine complex interrelationships among behavioral, psychosocial, and clinical variables, and to identify factors that occupy central positions within the interrelated diabetes management system. This approach is useful in diabetes research because behavioral, psychosocial, and clinical factors often interact rather than operate independently. The primary aim of this study was to evaluate the interrelationships between patient demographics, behavioral and psychosocial correlates of T2D, and fasting blood glucose among people with T2D in DR.

## Methods

This cross-sectional study was conducted at the *Centro Regional Contra la Diabetes Nagua* (NRDC), a non-profit regional diabetes clinic in Nagua, Dominican Republic. The evaluation was developed collaboratively with clinic leadership, staff, and board members.

### Participants and recruitment

Adult patients (>18 years old) of the NRDC with a diagnosis of T2D who were independently ambulatory were eligible.

Patient participants were recruited through flyers posted in the clinic waiting area and brief informational announcements by clinic staff. All participants provided informed consent in Spanish. The study was approved by the Institutional Review Board at [university name omitted for peer review] and the Dominican Consejo Nacional de Bioética en Salud (CONABIOS), and conformed with the guidelines set forth in the Helsinki Declaration.

### Data collection procedures

Data were collected in summer 2025 using investigator-administered questionnaires delivered verbally in Spanish by a Dominican physician and nutritionist [author initials omitted for peer review]. Fasting blood glucose was measured via finger-stick capillary blood by the clinic nurse with the Prodigy Autocode Blood Glucose Monitor (Huntersville, NC, USA), which produces 90% of results within 15 mg/dL of the reference value (9). Although point-of-care capillary tests for fasting blood glucose is less accurate than standardized clinical measures such venous fasting blood glucose (10), it was selected for this study following consultation with clinic administrators, who preferred to avoid additional blood draws for participants.

### Survey instruments

#### Sleep quality

The Pittsburgh Sleep Quality Index (PSQI) was administered to evaluate sleep quality (11). The PSQI is a widely-used 9-item questionnaire to evaluate sleep quality and disturbances over the past month. It provides component scores and a global score on a scale of 0–21 with higher score indicating worse sleep with a cutoff of scores greater than 5 indicating poor sleep quality (12). The PSQI has been shown to be reliable and valid in Spanish-speaking populations (13).

#### Diet quality

The Global Diet Quality Project Diet Quality Questionnaire (DQQ) for Dominican Republic, a 29-item survey was administered to evaluate diet quality, which had been adapted using Dominican Spanish (14). The DQQ has been shown to have an 80-93% agreement with individual 24-hr recalls (15, 16).

#### Perceived stress

The Perceived Stress Scale – 10 (PSS) is a widely used 10-item questionnaire regarding perceived stress over the past month. Higher scores on the scale indicate higher levels of perceived stress. The PSS has been shown to be a valid and reliable measure of perceived stress in Spanish-speaking populations (17) and in a wide variety of countries (18).

#### Health related quality of life

The Health-Related Quality of Life – 14 (HRQoL) is a 14-question survey developed by the Centers for Disease Control and Prevention to assess perceived physical and mental health. The questionnaire includes questions on general health status, recent physically and mentally unhealthy days, activity limitations, and symptoms like pain, depression, and anxiety. The primary outcome for this study was the healthy days core module, indicating the number of days in the past month without poor physical or mental health, which is a valid measure of physical and mental health (19).

#### Physical activity

The Yale Physical Activity Survey (YPAS) was designed to assess the amount and type of a wide variety of physical activities, such as housework, yardwork, leisurely walking, and vigorous exercise, in older adults during a typical week (20). The primary outcome is total weekly energy expenditure, with subscale values for the work, yard work, care taking, exercise, and recreation domains, with higher values. This questionnaire was chosen to in part because of its ability to differentiate various types of physical activity. The YPAS outcome, kcal•week^-1^ was used to estimate METminutes•week^-1^ using the formula: METminutes•week^-1^ ≈ kcal/week × 57.14​/body weight (kg) to compare to normative data (21).

### Data analysis

#### Network analysis pre-processing

All surveys were downloaded and scored in Excel and the calculated scores were used for the primary analysis. A Shapiro-Wilks test and histograms were used to assess normality of distribution. Due to the non-normal distribution of the data, a nonparanormal transformation was performed to approximate Gaussian distribution to preserve rank-order information. Binary variables were retained in their original form, and missing data were handled using complete-case analysis.

#### Sex comparisons

Due to the imbalance data (males = 17, females =48) and the heteroskedastic distribution in data, sex differences were assessed using independent-samples permutation tests. Permutation testing was selected because it is a non-parametric approach that does not rely on assumptions of normality or homogeneity of variance and provides robust inference for continuous variables. For each variable, the observed difference in group means (males – females) was compared with a null distribution generated from 10,000 random permutations of the group labels. Two-sided permutation p-values were calculated as the proportion of permuted mean differences that were as extreme as or more extreme than the observed mean difference. To account for multiple comparisons across all variables, Bonferroni-adjusted p-values were computed by multiplying the unadjusted permutation p-values by the number of comparisons with adjusted values capped at 1.0. Descriptive statistics (mean, standard deviation, and sample size) for each sex are also reported.

#### Primary network analysis

A sparse conditional dependence network was estimated using a Graphical Least Absolute Shrinkage and Selection Operator (Graphical LASSO). The improve robustness and reduce the likelihood of spurious associations, a bootstrap stability selection procedure was implemented. Specifically, 200 bootstrap samples were generated by resampling participants with replacement. Within each bootstrap sample, variables were standardized using z-scores and a Graphical LASSO model was fitted using 5-fold cross-validation to select the optimal regularization parameter. The resulting precision matrices were used to identify conditional associations between variables after controlling for all other variables. A Graphical LASSO was selected because it estimates sparse partial-correlation networks, facilitating identification of direct relationships while minimizing overfitting in high-dimensional settings.

For each pair of variables, the frequency with which an edge appeared across successful bootstrap iterations was calculated. Stable edges were defined as those present in at least 75% of successful bootstrap models. For retained edges, the mean signed precision coefficient and mean absolute precision coefficient were calculated across bootstrap iterations. Positive coefficients indicated direct positive associations, whereas negative coefficients indicated inverse associations.

The final network was visualized using Kamada-Kawai layout. Nodes represented variables and edges represented stable conditional associations. Only conditional associations with stability scores ≥ 0.750 were included.

Community structure within the network was identified using a greedy modularity optimization algorithm. Communities were interpreted as clusters of variables exhibiting stronger within-group than between-group connectivity.

To characterize the relative importance of a variable within the network, degree centrality was calculated as the proportion of direct connections association with each node. In addition, bridge degree centrality was computed to quantify the extent to which variables connected distinct conceptual gapes (predictors, control variables, and fasting blood glucose). Variables with higher bridge centrality were interpreted as potential connectors linking otherwise separate domains of the network.

All analyses were performed in Python using the pandas, NumPy, scikit-learn, NetworkX, SciPy, and Matplotlib libraries. Statistical inference was based on network stability rather than conventional null-hypothesis significance testing.

## Results

### Participant characteristics

Sixty-five (48 women; 17 men) people with T2D completed the questionnaire study. One participant was excluded from network analysis due to incomplete questionnaire data. Demographic, behavioral, and psychosocial results can be found in **Table 1**. Only 23% of participants were within the fasting blood glucose target range for controlled diabetes (80–130 mg/dL). Men had significantly higher fasting blood glucose concentration than women (243 ± 93 vs. 170 ± 64 mg•dL^-1^, p<0.001) and significantly lower physical activity (2035 ± 1161 vs. 2792 ± 1158 kcal•week^-1^, p=0.024).

**Table 1:** Demographic, behavioral and psychosocial characteristics of participants (mean ± SD or freq (%).

Variable	Women (n=48)	Men (n=17)	All (n=65)	Bonferroni-adjusted p-value (women vs. men)
Age (years)	58.1 ± 12.3	59.5 ± 12.6	58.5 ± 12.3	0.70
Body Mass Index (kg•m^-2^)	28.2 ± 4.9	27.4 ± 3.1	28.0 ± 4.5	0.46
Years since diabetes diagnosis	8.5 ± 7.4	10.1 ± 7.3	8.9 ± 7.4	0.46
Fasting blood glucose (mg•dL^-1^)	169.9 ± 63.6	242.8 ± 92.5	189.0 ± 78.5	*<0.001*
Met target fasting blood glucose (n, % total)	14 (29%)	1 (6%)	15 (23%)	–
Pittsburgh Sleep Quality Index (0-21)	10.9 ± 3.2	10.4 ± 3.0	11 ± 3	0.56
Diet Quality Questionnaire Global Dietary Recommendations (0-18)	9.5 ± 1.6	9.4 ± 1.9	9.5 ± 1.7	0.86
Perceived Stress Scale (0-40)	22.7 ± 5.2	23.2 ± 2.9	22.8 ± 4.7	0.71
Health Related Quality of Life Healthy Days (0-30)	18.1 ± 12.0	13.0 ± 11.7	17 ± 12	0.14
Yale Physical Activity Survey Energy Expenditure (kcal•week^-1^)	2792 ± 1158	2035 ± 1161	2594 ± 1198	*0.024*

MET, Metabolic Equivalent of Task.

ACSM, American College of Sports Medicine.

#### Sleep quality

When applying the standard cutoff score of greater than 5 indicating poor sleep quality, all but one participant (98%) reported poor sleep quality, with 57 (88%) having scores of 8-18, indicating moderate-to-severe sleep disturbances (22).

#### Diet quality

The mean Global Dietary Recommendations score of 9.5 ± 1.7 indicated low-moderate likelihood of meeting global healthy dietary requirements. The mean Non-communicable Disease Protect subscale was 1.9 ± 1.3 on a scale of 0-9, mostly due to diets low in fruits, vegetables, legumes and whole grains; nearly one-third of participants (32%) had consumed no fruits or vegetables during the previous day.

#### Stress

Only two participants (3%) were classified as low stress (Range: 0-13). All others were classified as moderate- (n=53; 82%) or high-stress (n=10; 15%).

#### Health related quality of life

The mean number of healthy days reported was 17 ± 12, meaning that participants were limited by poor physical or mental health on 13 out of the last 30 days, on average. Additionally, for perceived general health status (Poor – Excellent), about 75% of participants reported fair health, while no participant reported Excellent or Very Good health status.

#### Physical activity

The American College of Sports Medicine recommends greater than 500–1000 METmin•week^-1^ of PA to maintain health. In this sample, 54 (83%) participants completed more than 1000 METmin•week^-1^, and only 3 (5%) were below the 500 METmin•week^-1^ recommendation. The primary contributor to total physical activity was in the work domain (66%), with exercise (13%), recreation (11%), and yardwork (10%) being much smaller contributors. Within the work domain, the primary contributors were food preparation (19%), laundry (15%), shopping (13%), and heavy housework (11%), which are all between 2 and 4 MET activities, indicating that most of the reported physical activity fell within the light-intensity and low end of the moderate-intensity physical activity spectrum.

### Network analysis

The network analysis identified 8 stable edges among 10 variables (**Figure 1**). Community detection revealed four distinct clusters: (1) age, healthy days, and sleep quality; (2) fasting blood glucose, physical activity, perceived stress, and male sex; (3) Diet quality and years since diagnosis; and (4) Body mass index as an isolated node. Physical activity demonstrated the highest degree centrality (0.444; **Table 2**), indicating that physical activity was the most highly connected variable in the network.

**Figure 1:**
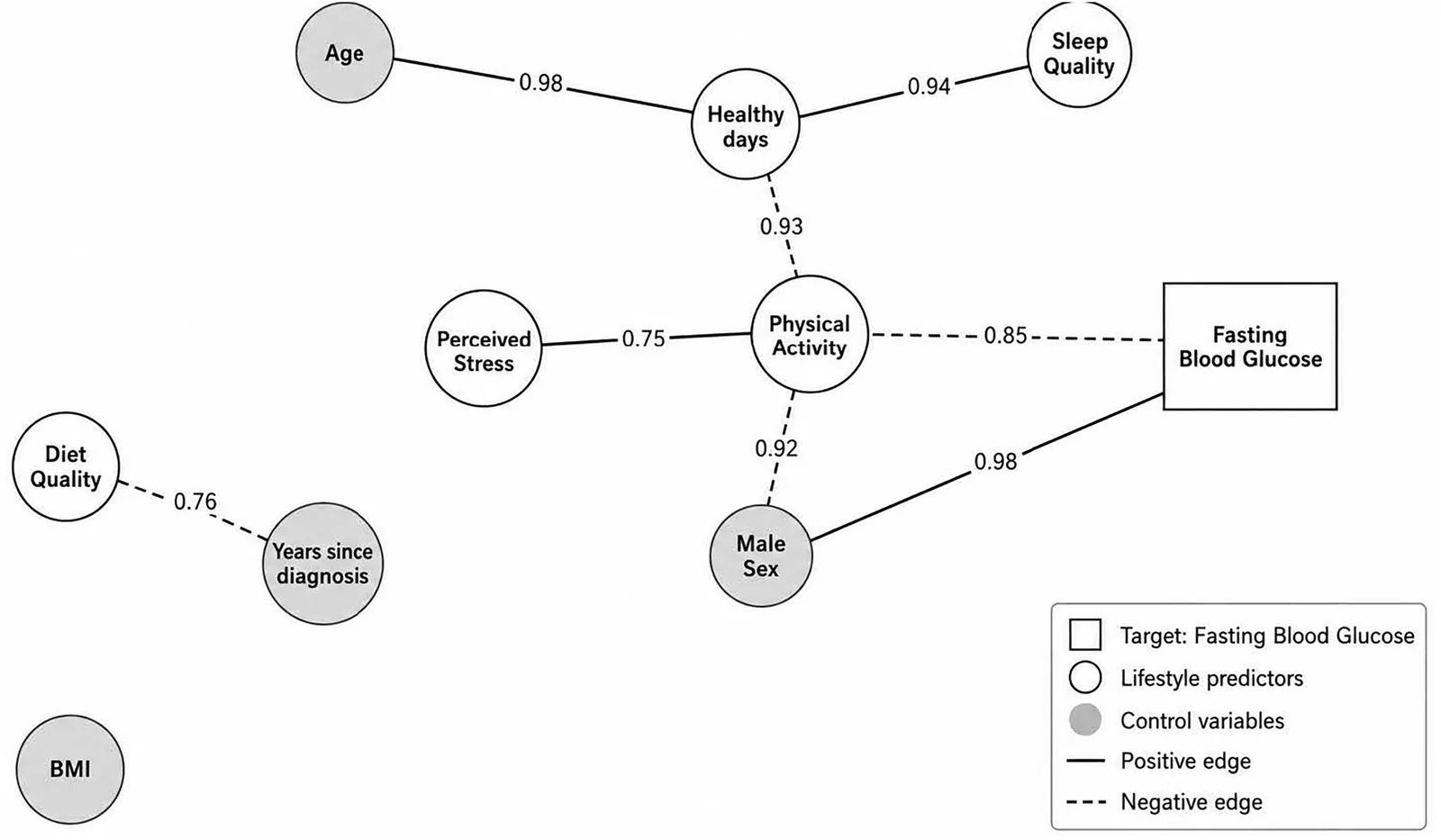
Network of demographic, behavioral, psychosocial, and clinical factors associated with fasting blood glucose among adults with type 2 diabetes in the Dominican Republic. Nodes represent study variables and edges represent stable conditional associations after controlling for all other variables in the network. Positive associations are represented by solid connections and negative associations by dashed connections. Four communities were identified: (1) age, healthy days, and sleep quality; (2) lasting blood glucose, physical activity, perceived stress, and male sex; (3) diet quality and years since diagnosis; and (4) BMI. Physical activity demonstrated the highest degree centrality and bridge centrality within the network.

**Table 2:** Network analysis metrics.

Variable	Group	Community	Degree centrality	Bridge centrality
Physical activity	Predictor	2	0.444	0.400
Healthy days	Predictor	1	0.333	0.000
Male sex	Control	2	0.222	0.333
Fasting glucose	Outcome	2	0.222	0.222
Sleep quality	Predictor	1	0.111	0.000
Perceived stress	Predictor	2	0.111	0.000
Diet quality	Predictor	3	0.111	0.200
Age	Control	1	0.111	0.167
Years since diagnosis	Control	3	0.111	0.167
Body mass index	Control	4	0.000	0.000

Higher degree centrality values indicate greater connectivity within the network. Higher bridge centrality values indicate greater importance in linking distinct communities of variables.

The most consistently retained association involving fasting blood glucose was its positive connection with male sex (stability = 0.98; **Table 3**). Physical activity was the only behavioral variable directly connected to fasting blood glucose, with an inverse association retained (stability = 0.85). Physical activity was also inversely connected to healthy days (stability = 0.93) and male sex (stability = 0.92), and positively connected to perceived stress (stability = 0.75). The inverse association between physical activity and male sex indicated less physical activity among men than women. Body mass index had no stable connections and formed an isolated community.

**Table 3:** Stable conditional associations identified in the network analysis.

Variable 1	Variable 2	Edge stability	Direction
Male Sex	Fasting blood glucose	0.98	Positive
Age	Healthy days	0.98	Positive
Sleep Quality	Healthy days	0.94	Positive
Physical Activity	Healthy days	0.93	Negative
Physical Activity	Male sex	0.92	Negative
Physical Activity	Fasting blood glucose	0.85	Negative
Diet Quality	Years since diagnosis	0.76	Negative
Physical Activity	Perceived Stress	0.75	Positive

Only stability scores ≥ 0.75 reported.

## Discussion

Patients demonstrated poor fasting blood glucose, particularly men, as well as poor sleep quality, moderate-to-high stress, poor-moderate diet quality and health-related quality of life, and predominantly low-to-moderate intensity occupational physical activity. An important finding was the disconnect between physical activity volume and fasting blood glucose. Although most participants exceeded recommended physical activity guidelines, nearly all physical activity came from occupational and household tasks that are performed at light-to-moderate intensities, suggesting that total physical activity volume alone may be insufficient to optimize fasting blood glucose. Greater emphasis on structured moderate-to-vigorous physical activity may be warranted to improve fasting blood glucose, but further prospective interventions are necessary to confirm any causal relationship.

In the current study, low-to-moderate intensity physical activity was the only behavioral or psychosocial factor associated with fasting blood glucose. However, because potentially important clinical and socioeconomic confounders were not included in the network analysis, this association should not be interpreted as evidence of an independent effect of physical activity on fasting blood glucose. Additionally, because fasting glucose was measured using capillary testing during a single assessment, the reported network association should be interpreted as an exploratory relationship with the measured fasting glucose value rather than evidence of an association with longer-term glycemic control. These findings are in line with previous research regarding the well-known benefits of physical activity for people with T2D (23), even when the intensity is relatively low (24). Surprisingly, physical activity was inversely associated with healthy days and directly associated with perceived stress, indicating that participants who experienced fewer healthy days and more perceived stress were more physically active. These associations need to be studied further in more heterogeneous groups to determine their relative strength and durability across wider population segments.

The fasting blood glucose concentrations observed in the current study were generally consistent with those reported in previous Dominican studies (6). More than 75% of participants had fasting blood glucose above target levels for controlled diabetes, with significantly higher values among men. Compared to prior Dominican studies reporting mean fasting glucose between 142–170 mg/dL (25–27), results in this sample appeared worse. These comparisons should be interpreted with caution, however, because fasting glucose was assessed using point-of-care capillary testing rather than standardized venous fasting glucose or HbA1c, which may have introduced measurement variability that could have affected both classification of glycemic control and the magnitude of observed group differences. Future research should be conducted to determine whether differential sex-specific interventions are warranted using venous blood draws to ensure validity of blood glucose data.

Nearly 90% of participants reported moderate-to-severe sleep disturbances, indicating a meaningful behavioral concern. Sleep duration and quality are important modifiable risk factors in T2D management (28); the very high prevalence of sleep disturbances in this sample supports the inclusion of sleep education and training in lifestyle interventions for people with T2D. Diet scores indicated low consumption of protective foods such as fruits and vegetables, which is consistent with the previous findings across the Dominican Republic (29). Future interventions in this population should include diet education to increase consumption of healthy-promoting foods for people with diabetes to improve diabetes management (30). Stress levels were moderate-high in this study. There may be a dose-response relationship between stress and glycemic control (31), with moderate stress being related to an 85% increase in poor glycemic control and high stress related to a 184% increase, indicating that even moderate stress reduction may have a profound impact on glycemic control.

Health-related quality of life findings were consistent with prior Dominican studies reporting moderate impairment among people with T2D (25, 27), although comparison is difficult due to different questionnaires having been administered. Although total PA was high, PA was predominantly occupational and low-to-moderate intensity, corroborating prior accelerometry findings from the same clinic (7) and low exercise reported in other studies of Dominicans with T2D (25, 26). Given the evidence that vigorous activity confers significantly greater metabolic benefit than moderate-intensity physical activity (32), additional structured exercise opportunities may complement existing high occupational PA in people with T2D.

### Limitations

This study had several limitations. Although the findings generated several hypotheses, the cross-sectional design did not allow for causal inferences from the findings. Low health literacy may have limited comprehension of the validated Spanish-language instruments, although they were verbally administered. Additionally, physical activity and other questionnaires included self-reported data to a health professional and therefore may have been subject to recall or social desirability bias. Blood glucose was assessed using fasting capillary blood glucose rather than fasting venous plasma glucose or HbA1c, which are used during standard clinical practice. Although point-of-care capillary testing was feasible in this resource-constrained clinical setting, it may have introduced greater measurement variability compared to laboratory-based testing, potentially resulting in misclassification of participants near the target fasting glucose range. Additionally, this fasting glucose measurement reflected glycemic status at a single time point, whereas HbA1c would have provided a better estimate of long-term glycemic control. Therefore, the glucose measure used in this study may have influenced the reported associations between fasting blood glucose and the behavioral and psychosocial factors observed in the network. Future studies should incorporate standardized venous glucose measurements and/or HbA1c when feasible. This study was conducted in a single regional diabetes clinic and included substantially more women than men, which limited sample heterogeneity and may restrict the generalizability of the findings. Additionally, potentially important clinical and socioeconomic factors, including medication dosage and adherence, comorbidities, and socioeconomic factors, were not included in the network analysis. Residual confounding from these factors may have influenced the observed associations, particularly those between physical activity, sex, and fasting blood glucose. The network relationships reported in this study should be interpreted as exploratory associations rather than independent effects, and confirmed with more robust follow-up studies.

## Conclusion

In this sample from a resource-constrained clinic in the Dominican Republic, fasting blood glucose was above the recommended target range. Additionally, participants reported poor sleep, moderate stress, suboptimal diet. Physical activity, which consisted of primarily low-intensity occupational and household activities, was relatively high, indicating that total activity volume alone may not adequately characterize physical activity relevant to diabetes management. In the exploratory network, physical activity was the only behavioral factor directly associated with fasting blood glucose, suggesting that total activity volume alone may not adequately characterize physical activity relevant to diabetes management. Higher intensity physical activity promotion may represent a promising target for future diabetes self-management interventions in resource-constrained settings, although its independent relationship with glycemic control should be confirmed in larger, more heterogeneous samples with greater control of clinical and socioeconomic confounding variables. These findings support the need for prospective studies to determine the effectiveness of multifaceted low-cost, low-burden interventions addressing both structural and behavioral determinants of diabetes outcomes in LMIC. Prospective studies using longitudinal network approaches may help determine how highly connected behavioral factors may predict the effectiveness of interventions to improve glycemic control in people with T2D. Future studies should incorporate objective physical activity measures and glycated hemoglobin, as well as include larger multisite samples.

## Data Availability

The raw data supporting the conclusions of this article will be made available by the authors, without undue reservation.
